# Molecular and macromolecular electrochemistry: synthesis, mechanism, and redox properties

**DOI:** 10.3762/bjoc.18.158

**Published:** 2022-10-26

**Authors:** Shinsuke Inagi, Mahito Atobe

**Affiliations:** 1 Department of Chemical Science and Engineering, Tokyo Institute of Technology, 4259 Nagatsuta-cho, Midori-ku, Yokohama 226-8502, Japan; 2 Department of Chemistry and Life Science, Yokohama National University, 79-5 Tokiwadai, Hodogaya-ku, Yokohama 240-8501, Japan

**Keywords:** electron transfer, electrosynthesis, organic electrochemistry, redox-active materials

Electrochemistry is now a powerful tool in organic chemistry not only for analyzing the electron transfer behavior of organic molecules and macromolecules, but also for driving organic reactions to produce value-added products. Electrochemical synthesis (or simply electrosynthesis) is increasingly recognized for the high academic and industrial importance, in line with the concept of green chemistry proposed in 1998 and the Sustainable Development Goals (SDGs) adopted by the United Nations in 2015. This is evidenced by the recent publication of special issues on organic electrosynthesis in various academic journals [1–5].

In addition to the conventional two- or three-electrode batch-type electrolytic cells, recent developments include microflow electrolytic reactors, polymer electrolyte membrane electrolysis technology, and new methods coupled with photoredox catalysts or transition metal catalysis, resulting in remarkable progress in organic electrosynthetic processes. Theoretical calculations have also led to a better understanding of the electron transfer behavior of organic molecules and the estimation of subsequent reactions, resulting in a much better understanding of the reaction mechanism. Furthermore, because organic electrosynthesis requires the setting of many complex parameters, such as applied potential, current density, electrolyte, temperature, and so on, it has a high affinity to informatics approaches, e.g., machine learning, which is expected to become an increasingly important tool in the future.

Progress in the design of organic molecules and polymers and the understanding of the redox behavior of these compounds has led to the development of organic electrochemistry for energy material applications. Organic semiconductor design for electron or hole transport is important for transistor and solar cell applications, and redox-active (but stable) organic and polymeric materials are promising for secondary batteries and redox flow batteries. By understanding organic electron transfer reactions, we can face the challenge of how to design materials with better cycle properties by suppressing undesired side reactions.

To showcase this area of research, the present thematic issue focuses on the recent advances in molecular and macromolecular electrochemistry. The scope of this interdisciplinary issue ranges from synthetic aspects (such as electrosynthesis and reaction mechanisms) to materials science (including redox properties and devices).

Shinsuke Inagi and Mahito Atobe

Yokohama, October 2022
